# Observations on the Surgical Pathology of the Larynx and Trachea

**Published:** 1827-04

**Authors:** 


*Observations on the Surgical Pathology of the Larynx and Trachea, chiefly with a View to Illustrate the Affections of those Organs which may require the Operation of Bronchotomy; including Remarks on Croup, Cynanche Laryngea, Foreign Bodies in the Windpipe, Wounds, &c. &c.* By WILLIAM HENRY PORTER, A.M. Surgeon to the Meath Hospital, and County Dublin Infirmary, and to the Dublin General Dispensary; Lecturer on Anatomy, &c. 8vo, pp. 283. Dublin and London, 1827.

THE greater part of the work before us formed a portion of a course of clinical lectures delivered by the author in the winter of 1825; and Mr. Porter modestly observes that his sole object in publishing these lectures, is to facilitate the student's progress, and help him to found his future practice on pathological principles, rather than on the unexplained results of numerous cases, the details of which can only burden the memory without ever improving the mind. But we are very much mistaken if Mr. Porter's volume will not prove deserving the attentive perusal of every practitioner, who is anxious to have his ideas well arranged, and his mind prepared for the treatment of a class of diseases that often come upon him with such suddenness and formidable symptoms as to leave him little time to search for information, or fortify his judgement by the help of precedents scattered in various works. Every one knows the importance of diseases which interfere with the functions of the respiratory organs, and the intense anxiety which such diseases create, not only in the patient, but in the bye-standers. To this may be added the fact that, in no case can the surgeon more completely or more decidedly display the efficacy of his art, than in those where he is sometimes enabled to snatch the sufferer from a state of fearful distress and danger, and restore him to one of comparative tranquillity. We allude, of course, to tracheotomy—an operation which early attracted notice, and which has, in modern times, been crowned with the most brilliant success. Mr. Porter has not found any attempt made to arrange those diseases, requiring such an operation, in pathological order—to point out the morbid appearances on dissection—and to connect these with their characteristic symptoms during life.

Whatever may be the case in other operations, Mr. Porter is of opinion that bronchotomy is too seldom practised, and that, when it is performed, it is generally at a period of disease when its success is impossible. Much incertitude prevails in this branch of surgery, and the descrepancy of opinion must have some cause. The aerial conduit, simple as it is in structure, is subject to a great variety of morbid affections, the symptoms of which have much resemblance to each other—some being remediable—some requiring manual operations—and many being beyond the power of art, even as to palliation.

Our author commences his work with a brief view of the phenomena attendant on mucous inflammations (that is, inflammations of mucous membranes) in general, and of the consequences resulting from them. After the pains which we have taken to spread information on these particular subjects, it will not be necessary to dwell much on them here. An extract or two, to shew the author's manner, and which may, at the same time, be interesting, will be sufficient. After giving a neat and a very correct sketch of the anatomical characters of mucous surfaces, in a state of phlogosis, Mr. Porter offers the following observations.

"In reference, however, to the larynx and trachea, it is certain that inflammation will produce purulent expectoration. Far, however, from this secretion being in itself a dangerous symptom, its appearance usually indicates the subsidence of the first inflammatory action, for it is when the vessels have begun to unload themselves,—when the intense redness has disappeared and the swelling subsided, that the purulent discharge appears to flow from the surface of the membrane. It would, in fact, be always a favorable symptom except that mucous surfaces, when once they have taken on any particular action have a strong disposition to continue it to an indefinite period. Gleety discharges form a good illustration of this tendency to the maintenance of a morbid action, and gleets from the pulmonary membrane would perhaps prove as trifling and afford as little trouble as those in any other situation, if it was not that the efforts to expectorate the secretion through the narrow aperture of the larynx very constantly produce such derangement in the lungs as to cause the most formidable symptoms and often a fatal termination. Indeed, it is frequently extremely difficult to distinguish between the protracted chronic forms of bronchial inflammation and regular phthisis arising from tubercular suppuration, for both diseases exhibit the cough, the nocturnal perspirations, the difficulty of lying on one particular side, and the purulent expectoration. And the similitude may be carried farther, for both very commonly have a like termination, the one dying of hepatized lungs, the other of the suppuration and destruction of the organ, as is observed in tubercular phthisis pulmonalis.

"A very common consequence of inflammations of mucous membranes is, that their sensibility is so altered that they ill endure the presence of those substances, for the transmission of which the organs of which they form a part were originally intended, and they certainly do not operate on them those changes usually performed during health. If the lining membrane of the bladder be inflamed, the viscus will not contain a drop of urine even for a moment, and the patient is perpetually teazed with the most urgent calls to expel this fluid. If the urethra be the seat of disease, the passage of the urine over the affected spot causes a sensation of burning pain that is almost intolerable. If the stomach is attacked, not only is the presence of food within it painful and disagreeable, but vomiting is a symptom constant almost without an exception: and it is well known if the intestines are inflamed, as in acute dysentery, that the smallest quantity of mucus is so painful and so irritating, that the patient is frequently called upon to discharge it. In applying this pathological observation to the membrane covering the larynx and trachea, two facts of some importance come before our notice. First, that the passage of the air is more or less accompanied by pain, for at every inspiration the patient shows evident symptoms of suffering, although his anxiety to inflate his lungs, and the horror that always accompanies the dread of suffocation, makes him forget the actual uneasiness, in his struggles to procure a sufficient supply of air. And, secondly, that in acute cases of bronchial inflammation, the arterialization of the blood is but very imperfectly performed." 17.

Inflammation of the aërial surfaces interferes so much with the important process of sanguification, or at least the change effected in the blood while passing through the lungs, that death is often produced by the interruption of this process, independent of any effusion into the bronchial tubes or air-cells.

"It is also remarkable, that cases of very acute bronchial inflammation are rapid, if not sudden in their termination. Sometimes, it is true, the patient perishes in consequence of a rupture of some of the air vessels of the lungs, caused by the violence of coughing, and the organ thus becoming emphysematous, and incapable of performing its office. Sometimes, perhaps, and particularly in aged subjects, a vessel of the brain may give way from a similar cause. On other occasions the mucous membrane is found partially smeared over with a substance resembling thin paste or honey, which might slightly interfere with its healthy functions; but in the great majority of cases I have had an opportunity of examining, the mucous surface appeared red, swollen, and pulpy, rather exhibiting a deficiency of its accustomed secretion, and unaccompanied by any other morbid production, unless a small quantity of serous effusion in the bronchial cells. This serous effusion I have just now noticed is found in greater or less quantity in all subjects that have died by obstructed or imperfect respiration, and has by some been considered as the ordinary product of mucous inflammation. But, independent of the circumstance that such fluid is not observed to be poured out by mucous surfaces in other situations, it is easy to account for its presence on other principles more consonant to the phenomena resulting from disordered breathing. Whenever, either by reason of an insufficient supply of air, or of the presence of inflammation in the membrane, the blood is but partially or imperfectly arterialized, congestion is the consequence, and the vessels, to relieve themselves, begin to pour out their serous contents. If the original cause be longer maintained, the heart is obliged to supply the brain with a fluid inadequate to maintain its healthy tone and actions, and the nervous influence imparted to the whole body, and of course to those parts concerned in the important process of respiration, being impaired, the lungs become still more loaded, and the effusion is continued and increased. Finally, the energy of the brain fails, and the muscles of respiration cease to act: and thus the patient dies, not because an effusion of serum into the bronchial cells had caused suffocation, but because inflammation had prevented the arterialization of the blood, and thus deprived the brain of that stimulus which is necessary to the maintenance of its healthy actions." 20.

Ulceration is a very frequent consequence of mucous inflammation generally; but less so in the larynx and trachea than in other situations. Mr. P. divides these ulcerations into three forms—*first*, those which take place at the apertures of mucous canals, assuming the appearance of ragged cracks, spreading irregularly, scabbing, and healing without granulation. *Secondly*, Aphthous ulcers so well known, and generally dependent on irritation in the stomach or bowels. These do not appear to heal by granulation, but by a gradual contraction and approximation of their edges. The *third* form is the most important.

"Then we have a broad, flat sore, sometimes deep, sometimes superficial; its surface covered with a tenacious slough of a bright yellow colour, and its edges uneven, if not ragged. These sores have a wonderful disposition to spread, and frequently we see them rapidly making way in one direction, whilst they are healing just as rapidly in another. They are often met with on the surface of the intestines of scrofulous children, but their most frequent situation is in the throat, where, commencing at the back of the pharynx or on the edges of the arches of the palate, they spread downwards to the larynx, and produce the most formidable consequences. The soft palate is often altogether destroyed by them, and perhaps, after continuing during an immense length of time, and resisting every application and all kinds of medical treatment, they seem to heal spontaneously; but in general their termination is not so favorable. I believe they are most commonly fatal; but death is not induced either by the irritation they create or the ravages they commit, but by the general constitutional derangement to which they owe their origin, and of which they may be considered in the light of symptoms. The most perfect specimen of this form of ulceration I ever witnessed was in the case of a man who died of laryngeal disease in the Meath Hospital. He was of a highly scrofulous disposition, had contracted a venereal taint, and taken mercury irregularly, so that he seemed to have suffered from a combination of morbid excitements at the same time, under which his health rapidly broke down. On examination after death, a number of broad, flat, irregular ulcerations were found in the œsophagus, with ragged, uneven edges, and their surfaces covered with a tenacious matter of a bright gamboge-yellow colour." 22.

The sloughs and croupy exudations which form on mucous membranes are well known. Mr. P. touches on the subject of spasm of mucous canals, which may happen without, but more frequently *with* inflammation, adding considerably to the patient's sufferings, and frequently proving the cause of death. The spasm, of course, is actually seated in the fibrous structures surrounding mucous canals, rather than in the mucous tissues themselves. Whatever can excite these muscular structures into inordinate action, will occasionally give rise to spasm. Sometimes it appears to be a sympathetic affection, as in the spasmodic croupy respiration of children, while under the irritation of teething or of deranged digestion, &c.

After these and many other general observations on affections of mucous membranes, our author proceeds to a more particular investigation of those diseases which appear limited to the larynx and trachea, and which occasionally demand surgical interference—more especially bronchotomy. These are, 1. Acute inflammation of the mucous membrane in the child, constituting croup, or cynanche trachealis—2. Spasmodic croup, without inflammation—3. Inflammation of the submucous tissue of the larynx in the adult, or acute cynanche laryngea—4. Thickening of the membrane, or chronic cynanche laryngea—5. Alteration of structure in the laryngeal cartilages, or phthisis laryngea—6. Sloughing and death of the cartilages —7. Pressure on the air passage by abscess, tumour, or other affection. To the above may be added, 8. Those affections of the larynx arising from accident, as the swallowing boiling water, the admission of foreign bodies, and injuries or wounds, as in attempts at suicide.

### I. CYNANCHE TRACH: OR CROUP.

Laying aside the vague and arbitrary denominations and divisions of croup, our author founds his classification on dissection, and on this plan divides the disease into three species: —One where dissection shews no appreciable lesion of structure, nor the remains of any increase of action—the larynx and trachea appearing pale, smooth, and polished, without any indication of vascular action or thickening of the membrane. In such cases, the occurrence of death can only be explained by supposing the existence of some irregular action, as spasm. This form is by no means infrequent—is often fatal—its attacks are sudden—and it carries off its victim with great rapidity.

The *second* form constitutes the inflammatory croup, exhibiting, on dissection, all the appearances and all the stages of acute inflammation of the bronchial membrane in the child. It is this form of disease which produces the adventitious membrane of coagulable lymph, and it is by far the most frequent of all the forms. When acute in its symptoms it is very generally rapid and fatal.

The *third* form is very rare, comparatively speaking, as our author has only met with two examples out of a great number of cases. It is where the lining membrane of the larynx has become altered in structure and thickened, so as to interfere with the functions of the organ. The disease is insidious in its approach, slow in its progress, but inevitably destructive in the end. "It is comparatively common in the adult, where it constitutes one of the examples of chronic cynanche laryngea:"—or, in other words, laryngeal phthisis.*

Passing over the supposed causes of croup, which are very obscure, our author remarks that it is much more common among the rich than the poor, and thinks the reason of this is the pampered state of children in the upper classes of life. Mr. Porter confirms the observations of Bretonneau, Mackenzie, Gregory, Pretty, and other writers, respecting the occasional spreading of the inflammation from the fauces and tonsils to the larynx, there producing the inflammatory exudation. Ulceration also in the throat sometimes spreads to the glottis, and becomes the exciting cause of genuine croup. The symptoms of croup are fairly described by our author, and then the treatment comes under consideration. He complains that, hitherto he "has not been able to discover any attempt to arrange the treatment of croup with pathological precision," in consequence of which, some recommend bleeding, others blisters, the warm bath, emetics, mercury, &c. &c. Even bronchotomy has been recommended as a dernier resource, and success has sometimes crowned the operation. "But there is no form of the disease, or no period of its duration, attempted to be pointed out in which one remedy is more clearly indicated than another." Hence the young practitioner is left in doubt, and either selects by chance, or follows some favourite authority. This, we fear, is the case, and must always be the case, in more complaints than croup.

As, in the commencement, the disease is incipient inflammation, the indication is, of course, to subdue the morbid action, and prevent the production of an artificial membrane. After the exudation is formed, "then (if we possessed the means) the object should be to procure an artificial passage for the air, which would afford the double advantage of preserving the lungs from congestion, and allowing repose to the larynx, whilst, by the common process of nature, the membrane might be separated and expelled." The last stage of croup presents the brain oppressed with blood of an improper quality, and all the energies of the system consequently weakened. The result is then necessarily fatal, "even if free respiration

* In the adult our author must surely have seen more than two examples of this disease; since it is by no means rare. Indeed, it is much more common than is imagined; for many die of supposed phthisis pulmonalis, who fall victims actually to this form of disease.—*Rev.*

could be restored." The following passage is recommended to the attention of those who consider blood-letting all powerful in acute inflammations. We have often urged the same advice which is here conveyed.

"It will in general be found that cases of recovery in croup have been frequent in proportion to the early adoption of remedial measures, because it is one thing to check inflammation and prevent its effects from taking place, and another to remove those effects when they have been fairly formed; and because it is to the first of these objects that the treatment of croup has been directed. But it appears to me not to be sufficient to diminish an increased action, unless the constitution be kept, until the period of danger is over, in a condition that will render a renewal of that action unlikely to occur; for, however a patient may be brought down, (suppose by blood-letting,) if he is not kept in this state a re-action will take place, and a disposition produced in the system exceedingly favourable to the progress of inflammation. It is this, which causes even large bleedings to be so frequently inefficacious in the treatment of acute diseases, and renders a repetition of them necessary before the affection is subdued: whereas one operation of the kind, if followed by measures calculated to maintain a state of nausea or debility, will usually be sufficient.—In many inflammatory affections, such as those of the head or of the gastric organs, the exhibition of medicines possessing these powers is plainly impossible; but in those of the throat and chest similar objections do not obtain, and I have used them frequently with at least a fair proportion of success." 42.

General blood-letting in croup is preferred by our author to local, as it is desirable to make a direct and considerable impression on the system. On the use of emetics our author makes some good remarks, though he does not seem to hold them in very high estimation. When, however, the child is fat and plethoric—unaccustomed to exercise, and overfed—when he is suddenly attacked, perhaps after a hearty meal, it will be desirable to evacuate the stomach. "But there is this disadvantage attending the practice, that it will be next to impossible to administer nauseating medicines afterwards."

As croup is always attended by spasmodic exacerbations, and these, if not originally excited by intestinal irritation, are always considerably aggravated by it, so purgatives are indicated, but they are too slow in their operation. The same objection, Mr. P. observes, holds against blisters, which should never be applied in the vicinity of an inflammation. In the latter stages, where the lungs are threatened by congestion, they may be applied to the chest. Our author has witnessed the beneficial effects of mercury; "but its use is nearly confined to long-protracted and chronic cases, where there is a tendency in the mucous membrane to become thickened and changed in its structure." Sometimes in large doses it has appeared to be beneficial, even in the commencement of the attack, which our author is unable to account for.

The subject of bronchotomy as a remedy, is then taken up by our author, and the pros and cons fairly considered. After concisely stating the reasons adduced by the advocates for the operation, Mr. P. observes that there are many and serious objections to be urged against the practice. Although the effusion of coagulable lymph is generally confined to the larynx alone; still, in a number of cases, the "inflammation commences in the bronchial cells, and spreads upwards in the windpipe." Here the operation would be of no service; "and there is no mode of distinguishing accurately as to what has been the original seat of the disease."* This consideration, our author observes, must involve every case in obscurity, "and render the success of an operation a matter more dependent on chance than on judgment." This objection, as we have shewn in the note, is not so valid as Mr. Porter makes it appear to be. The stethoscope would clear away much of the uncertainty.

---

* Mr. Porter was not then acquainted with the use of the stethoscope on such occasions; but we find, in a subsequent part of the volume, a note which is of too much consequence to be passed over.

"At the time the above remarks were written, the same facilities for ascertaining the condition of the lungs did not exist that are enjoyed at present by means of the stethoscope. This instrument was scarcely known in Ireland, and like most other novel inventions, was decried by some and ridiculed by others. Amongst the rest, I confess I had very little faith in the accuracy of prognostics made by means of this tube, and as I had seen some of them erroneous, my conviction of its imperfection, if not of its inutility, was rather strengthened. Of late, however, I have been induced to alter my opinion very considerably. The stethoscope is receiving a full, fair, and impartial trial in the Meath Hospital by Doctors Graves and William Stokes, and it is quite clear from the accuracy of their observations during the life-time of a patient, and the strict accordance of those with the pathological investigations after death, that many of the objectionable circumstances connected with its early use arose rather from the inexperience of the practitioner than any decided imperfection in the instrument. Like most other improvements, it has probably been advocated too strongly on the one hand, and opposed on the other, on opinions too hastily formed; but it must be remarked that opposition has usually proceeded from those who understood least about the matter, whilst the more it has been cultivated the more warmly has it been supported. It is a mode of investigation that can only be learned by practice.—No description can convey the idea of one particular kind of wheeze or another, and as its use is not well accommodated to the bed-side of a private patient, it will be long before it comes into general use, except in hospitals." 102.

Here then is a testimony from one who acknowledges himself to have been sceptical as to auscultation.

The following objection is of greater weight. "If it be true, that inflammation interferes with the functions of the bronchial membrane, and that the blood will be imperfectly arterialized when such disease is present, it will be little consequence whether air be admitted or not—the brain will as surely be affected as if no artificial opening had even been practised, and all the relief the patient will experience, can amount to no more than a cessation of that *extreme muscular exertion* which is necessary to carry on respiration at all."

But surely Mr. Porter does not mean to say that the only inconvenience of an obstruction to the free entrance and exit of air to and from the lungs is merely an additional exertion of certain muscles. It is impossible to imagine any thing more torturous than what results from mechanical impediments in breathing; and were the operation of no other benefit than the relief of this part of the complaint for four hours before death, it would be well worth undergoing—at least we should prefer it to four hours of the agony in question. This temporary relief is thus exemplified :—

"I saw this admirably exemplified in the case of a little girl on whom bronchotomy was performed for the cure of croup: the disease had originally been confined to the larynx, but after the operation, the bronchial cells became affected, and the inflammation spread upwards nearly to the place in which the trachea had been opened. In this instance there was no deficiency of air: the aperture was much larger than the natural size of the rima glottidis, yet the patient had convulsions, exhibited every symptom of cerebral congestion, and finally died comatose." 49.

This comatose death is, of course, one of the easiest—and, therefore, on the principle of Euthanasia alone, we should be inclined to recommend the operation, when all other means failed. This, however, is not the opinion of the author, who presses into his reasoning certain *prudential* motives, which ought not often to interfere with the dictates of truth, science, and humanity, in surgical operations.

"In a disease that runs its course with such rapidity, it would be desirable to ascertain at what period the operation should be performed, and what are the symptoms that indicate its necessity. In the earlier stages, when the membrane is red and swollen, and no lymph as yet effused, there can be no object in making an incision which will be much more likely to aggravate the disease than to relieve it. When the adventitious membrane has been formed, there is some reason to think that in the great majority of cases sufficient mischief has been already accomplished to render a recovery very problematic. The lungs have been already loaded with blood: perhaps effusion has been begun, and it may be, from the irritation it has undergone that the mucous membrane of the bronchial cells has already taken on a disposition to inflammation. It may be that the brain has already become affected, for I have met with many instances in which the disease proceeded with such rapidity that no lymph has been effused, and yet the patients never during life shewed any symptom that could mark a difference between the two cases. At the latter stages of croup, it would be absurd to think that an operation could possibly prove beneficial, unless it be supposed that a wound of the windpipe could remove cerebral congestion; and therefore whenever convulsions have occurred, or that the patient appears *comatose or sinking*, let no man undertake it as a last resource, for it is a resource that will avail him little, and after his patient's death he may esteem himself fortunate if a great part of the blame is not laid to the account of himself and his knife." 50.

No man, of course, would think of performing bronchotomy when the little patient was dying *comatose*. His sufferings are then over, or nearly so; but it is before this state of things has occurred—when the agony is great, and the sensorial functions are unaffected, that the free opening would certainly mitigate the suffering, and could not, we believe, increase the danger.

Mr. Porter observes, that it will be necessary to draw a distinction between the struggles of a patient endeavouring to free himself from some obstruction in the windpipe, and the convulsions just alluded to; for, in both cases, the patient's countenance will become dark and purple, his eye suffused and staring, and his respiration heaving and laboured—and both these affections occur at a late period of the disease.

"If the inflammation is about to subside and to terminate in an extensive effusion of mucus, the patient's struggles to relieve himself from the oppression naturally created by the accumulation of fluid in the respiratory tube, must be not only distressing but injurious, and may prove fatal. In this case an operation which will afford a clear and easy passage for the fluid will probably be followed by the happiest consequences: or if there was reason to believe, that on the total subsidence of the inflammation, the membranous lymph existed in the trachea purely in the condition of a foreign body, an opening which would afford means for its easy expulsion might also be judicious. But it is unfortunate that no symptoms exist, by which a practitioner can positively establish the propriety of his operation; there are in both these forms of disease the same cough—the same difficult respiration—the same struggles to inflate the lungs and to free the air passages from the impediments that are within them. To these sources of difficulty may be added, that any obstruction to the air cannot endure long without the lungs becoming loaded and oppressed, and therefore if the operation is not performed almost at the exact minute between the subsidence of the inflammation and the commencement of the effusion, it will scarcely be successful. Thus it happens, that in the great majority of cases, when bronchotomy has been performed, the patient finds himself wonderfully relieved; great quantities of mucus are expelled by the wound; respiration is free, and a surprizing degree of tranquillity seems to be suddenly obtained. But the calm is only deceitful. In the course of two or three days the patient begins to sink. He is unable to expectorate the mucus which now accumulates in greater abundance than before. He becomes heavy and languid—is with difficulty roused from this state of stupor, and generally within four or five days after the operation he dies comatose." 52.

In cases of purely spasmodic croup, where there is reason to believe that no organic disease whatever had existence in the wind-pipe, "the operation might not only be resorted to at the moment of attack, but probably offers the means of resuscitation, if very speedily adopted subsequently to apparent death." The following brief, but correct sketch of this mysterious and quickly fatal affection may be deserving of extract.

"It is in general a diseased action consequent on irritation, and should be considered rather as a symptom than as a disease itself. It occurs in very young children,* and appears at the commencement of the period of dentition; sometimes earlier, and but very rarely after the child has reached its third year. The crowing noise which some infants make in respiration, and which nurses occasionally consider as a sign of thriving, is nothing more than the air rushing through the aperture of the glottis in a state of spasmodic constriction; and whenever this symptom appears, (however well the infant may seem in other respects) it is always advisable to pay attention to it, and remove the source of intestinal irritation which will generally be discoverable. In some instances the constriction is both slight and momentary, and the spasm never produces either unpleasant or dangerous consequences: in others it is longer continued, and the infant works and struggles for breath, becomes purple in the face, and is apparently dying when the spasm begins to relax, and a long-drawn, crowing inspiration, sets all things to rights again, and after a little time the patient revives, and recovers well. These results of spasmodic constriction of the larynx are often mistaken for true cerebral convulsions, and form no inconsiderable proportion of the fits said to be occasioned by teething. In the fatal cases of spasmodic croup death is extremely sudden: the infant may be apparently in good health, and in a moment he makes a violent effort to inspire, which occasions something like a faint scream or cry, becomes black and swollen in the countenance, and dies before assistance can be pro-

* "This is probably the disease which has been considered and described as cerebral croup, and on which Mr. Pretty has some excellent observations in a paper published in the Medical and Physical Journal, Jan. 1826, to which I beg to refer the reader."

cured. Indeed if a surgeon were standing by the patient's side at the moment, it is questionable how far his interference might prove serviceable." 54.

In this disease prevention is of more importance than the attempt at cure. Attention to the diet and secretions of children would almost invariably ward off such assaults; "but if the attack be severe, and the child is in imminent danger, will the surgeon be warranted in making a bold plunge with his knife into that portion of the wind-pipe between the thyroid and cricoid cartilage, in order to restore uninterrupted respiration?" Mr. Porter believes not. "But if the infant is, to all appearance, *dead*, and if the practitioner is called to him within any reasonable time, he should then, with the least possible delay, endeavour to inflate the lungs, and restore animation by whatever means shall appear to be the speediest, and of these perhaps the most preferable will be laryngotomy." Now we really think it is sacrificing rather too much to prudential motives, to allow an infant to expire of croup, and then begin the work of resuscitation, because no blame could be attached to the surgeon for an ineffectual operation on the dead body.* We do not, indeed, wish to inspire sanguine hopes from the operation of bronchotomy in croup, because we are afraid that the affection of the mucous membrane will generally be too far advanced before an operation would be permitted; but if it be consistent with sound practical knowledge that an operation were desirable, we think it ought to be proposed at the proper time, independent of all considerations of a prudential or personal nature, on the part of the surgeon.

We shall notice a few of the cases which are introduced by our author in illustration of this part of the work.

*Case.* Mary Flaven, five years of age, was suddenly seized with shivering, nausea, hot skin, and other symptoms of fever; and in the course of the night, with symptoms of croup, in a most violent degree. Next morning our author saw her, in a state of excessive restlessness—her breathing loud, harsh, and shrill, "indicating that the rima glottidis was nearly closed"—pulse very rapid—much fever—dry cough. The child could not speak so as to be understood. The inspirations were performed with almost convulsive exertion. She was bled—had an emetic—took James's powder with calomel—warm baths, &c. But all was in vain; she died the ensuing night, 35 hours from the commencement of the attack.

On dissection the mucous membrane of the larynx was found red,

---

* "The less a surgeon uses his knife on the neck of a child at this tender age, perhaps the better for *himself*." P. 54.

very much swollen, and somewhat œdematous in appearance. The surface of the membrane, both in the larynx and trachea, was covered with a yellowish soft substance resembling paste, which could be easily scraped off, when the inflammation was found extending down into the air-cells. There was some effusion of serous fluid into the trachea. This was the only case Mr. Porter had ever met with, in which the tumefaction of the mucous membrane of the larynx appeared to be sufficiently considerable to create any serious impediment to the passage of the air. The brain was very vascular, but no other organ affected.

*Case* 2. Miss E——, aged 4½ years, after a slight febrile attack, of a few days standing, was seized with symptoms of croup—the cough being hard and dry—the fever considerable—the tongue foul—and pulse 140. There was constant restlessness. This child was bled with leeches to a great extent, without appearing much debilitated; while emetics were exhibited internally; but nothing seemed for a moment to arrest the progress of the disease, and she died in 34 hours from the commencement of the attack.

On dissection, the larynx was found perfectly sound, except that there was some frothy mucus entangled in the ventricles. There was no trace of any adventitious membrane. Immediately below the larynx, the trachea was inflamed, red, and pulpy, the intensity of colour increasing as it proceeded downwards, to the air-cells. These and the trachea were filled with a serous fluid, of a reddish brown colour. The lungs did not collapse when the thorax was opened.

After some more cases with the dissections are detailed, our author relates five cases of croup successfully treated. The methodus medendi consisted almost exclusively in administering the solution of emetic tartar, (generally about an eighth of a grain for a dose,) so as to keep up nausea short of vomiting. In some cases actual vomiting occurred. We observe that only one out of the five patients was bled.

Two cases are next detailed, in which bronchotomy was performed. Of these we shall take some notice.

*Case* 3. An interesting little girl, about five years old, was attacked, on the 10th May, with troublesome dry cough. On the 11th some imperfection of voice appeared, with spasmodic difficulty of breathing, and increase of the cough. At this time it was mistaken for hooping-cough, by the first practitioner who was called in. On the 12th and 13th, the above-mentioned symptoms increased gradually, till the evening of the latter date, when an access of fever supervened, followed by dreadfully spasmodic difficulty of breathing, reducing the little patient to an alarming condition. On the 14th Mr. Hewson was called in, and determined on Bronchotomy, which was cheerfully submitted to by the little patient. When completed, "she appeared to be much relieved, and fell asleep, quietly breathing through a canula introduced into the trachea." On the 15th she was improving rapidly—"she was recovering strength, and able to sit up in bed." She was teased with the mucus expectorated through the wound. Her pulse had fallen considerably in frequency—"she had some appetite, and ate a little light food with apparent relish." Her sleep was frequently disturbed, however, by the mucus accumulating. On the 16th "she appeared to be still improving, and hopes were entertained that she would ultimately recover." On the 17th, at 4, p. m. she took a turn for the worse. The mucus rapidly accumulated, and she died at 12 o'clock that night.

*Dissection.* The mucous membrane of the larynx seemed entirely altered in structure, being of a yellowish opake colour, granulated, and having flocculi of lymph floating in the cavity of the organ. When these flocculi were detached, the membrane underneath appeared thickened and altered, but not red. There was no ulceration. The ventricles were obliterated, and the figure of the organ altogether spoiled. The boundary of the disease was abrupt, and confined to the larynx, terminating exactly at its inferior edge. The trachea was filled with mucus, frothy and reddish—and about half an inch below the situation of the wound, a blush of inflammation was perceptible, which increased in intensity as it descended lower in the tube. The mucous membrane of the bronchia was red, swelled, puffy, and lightly smeared over with a yellowish pasty substance.

This was a remarkable case—and certainly the relief afforded by the operation shewed that the chance of life was increased, not diminished, by that process. We conceive that the means of reducing inflammatory action were not carried on with sufficient vigour after the operation, to give this little patient all the advantages which the operation itself was capable of affording. It is not to be understood that bronchotomy will subdue bronchitis—it only affords time for nature and art to cure the disease; and if people look tamely on, and even give a patient food during the temporary relief afforded by the operation, they cannot expect much success.

*Case* 4. Emily Toole, about 3½ years of age, was attacked with croup, on the 10th June, but did not come under our author's care till a day or two had elapsed. The disease was very acute in its symptoms, and promised to be rapid in its progress. "The child's lips were already livid and purple—cheeks swollen and very pale—eyes prominent, white, and bloodless." The veins of the neck were sometimes greatly dilated, and the muscles of respiration almost convulsed. The pulse was very small, and too rapid to be counted—the cough incessant—the breathing hard and sonorous. The operation of bronchotomy was resolved on, without much prospect of success. The hæmorrhage was considerable, and the difficulty of the operation great, from the struggles of the child. It was half an hour before the trachea could be incised, for fear of the blood flowing into it, and suffocating the patient.

At length a small opening was made, when the child expelled some bloody mucus. "The difficulty of breathing was removed, and it fell fast asleep in half an hour afterwards."

In the evening, the child had rallied a good deal; "but it became apparent, that the operation had accomplished nothing." The cough was incessant, and the efforts to expectorate ineffectual. When mucus was brought up to the wound, it was sucked back by the next inspiration. She died in 12 hours after the operation.

On dissection, it was found that the larynx had been the seat of the disease, and was covered with a thick layer of adventitious membrane, which seemed nearly capable of blocking up the rima glottidis altogether. There was no trace of inflammatory action in the membrane around the incision. Just at the bifurcation of the trachea the membrane began to assume a red colour, which was continued downwards. The lungs seemed a little firmer than natural, and sections made into their substance gave issue to dark-coloured blood. There was considerable effusion into the bronchia and air-cells.

The state in which the child was, at the period of the operation, precluded much hope of success; but still we think the bronchotomy was justifiable.

### II. LARYNGITIS ŒDEMATOSA.

Our readers will find, in the 3d volume of this Series, (July, 1825) p. 205 to 210, an interesting paper on this subject from the pen of M. Bouillaud. It is but of late that the complaint has attracted the attention of pathologists, although, from the number of cases recently published, there can be no doubt that the disease is of very common occurrence. Passing over all speculation on the knowledge which the ancients are supposed to have had of laryngitis œdematosa, we may observe that the seat of this affection is more in the cellular tissue connecting the mucous membrane to the adjacent parts, than in the membrane itself, although the latter is often found to have been inflamed. The effect of inflammation in this sub-mucous tissue is an effusion of serous fluid within its cells, and a mechanical obstruction to the passage of air to the lungs through the rima glottidis. The danger of such a disease must therefore be in proportion to the quantity of effusion that takes place, and the rapidity with which it is formed, so that a patient may be quickly suffocated, or left to struggle three or four days with partially obstructed respiration, and finally congestion of the lungs and brain. The exciting causes appear in no wise to differ from those of inflammation in general—such as exposure to damp, to cold, to atmospheric transitions, &c. It is sometimes insidious, sometimes rapid in its approach. Our author knew two instances where young men had retired to bed at night, without complaining, and were found dead from this affection the next morning.

"The symptoms of acute cynanche laryngea might almost be enumerated from considering the morbid actions that have taken place, and the changes produced by them. They may, with reference to the treatment of the disease, advantageously admit of division into two classes: first, those merely indicating the existence of some mechanical obstruction which prevents the lungs from receiving a sufficient supply of air; and secondly, those shewing a state of congestion in the lungs, and perhaps in the brain.

"The former of these will present some variety, according as the disease may happen to be complicated with other inflammatory affections. In general its attack is sudden, but it may be otherwise; and if it be preceded or accompanied by cynanche tonsillaris, there will be previous shivering, nausea, headache, loss of appetite, heat and dryness of skin, with accelerated pulse, and other symptoms of inflammatory fever. Along with these, there will be a greater or less difficulty of deglutition, redness and swelling of the fauces, and enlargement of the tonsils. The occurrence of such symptoms as these may possibly lead the practitioner astray, if it should induce him to suppose that the difficulty of breathing arose from the inflammation of parts surrounding the larynx, and that the mischief was not situated within the organ itself. It is asserted on high authority,* that a combination of difficulty of deglutition with obstructed respiration, forms the essence of this disease; but it by no means follows that there should be any imperfection in the act of swallowing; for the larynx may be totally blocked up, so that scarcely a particle of air shall pass through it, and yet every part of the fauces be found after death to have been entirely free from disease.

"At first this affection makes its appearance with difficulty of breathing, and a sense of dryness or huskiness in the throat, which obliges the patient to cough frequently in order to get rid of what seems to be an extraneous source of irritation. This increases rapidly, and he is obliged often to draw a full inspiration in order to inflate the lungs, and in doing so there is a painful sensation of constriction about the windpipe. Soon the respiration becomes sonorous and laboured. There is a peculiar sound caused by the air forcing its way through the contracted aperture, which cannot be described, but which once heard, can never be mistaken. It is harsh, sibilous or whistling—accompanies each act of inspiration, and is less distinct, or perhaps wanting in expiration. The patient now becomes excessively anxious and uneasy: he has a strong disposition to slumber, and perhaps sleeps for a moment or two, but soon starts up in all the horrors of impending suffocation. His face is flushed; his eyes protruded, as if starting from their sockets; his lips

* "Sir G. Blane. Med. Chir. Transactions, v. 6."

swollen, but pale, and as if transparent; the larynx and trachea are moved quickly upwards and downwards in the neck, and all the muscles of respiration are brought into almost convulsive action, so that the chest heaves violently. At this time the patient cannot lie down, partly because the position is uneasy, and partly because he dreads falling asleep, and the horrible sensations with which he awakes. He will be generally found walking about, occasionally going to an open window with a view of inhaling purer air, and sometimes stopping to grasp a chair, or any other body which may serve to fix his arms, and thereby bring into action additional muscles to assist the process of respiration.

"In this stage the patient's voice is greatly impaired, but it is rather an inability to articulate at all than what is usually termed hoarseness. When asked as to the seat of his uneasiness, he points to the pomum adami. He is subject to dreadful spasmodic exacerbations, in which all the symptoms are aggravated, and the sweat pours off his forehead in abundance. The pulse at all times indicates the presence of irritation, being above 100, and occasionally 120, small, quick and vibrating.

"It would be difficult to determine, on seeing a case of this description, whether the symptoms arose from the actual presence of inflammation in the mucous membrane of the larynx, and its becoming consequently thickened and swollen and pulpy, or whether they were occasioned by the effusion of serum within the submucous tissue. Yet a material difference of practice would depend on such discrimination, if it could be accomplished, for if the disease be purely inflammatory, unaccompanied by any mechanical obstruction, the great probability is, that it will be relieved, and ultimately removed by blood-letting and other measures of depletion, whilst, if the effusion has taken place, the object will no longer be to check inflammation, but to remove certain effects of it which have already been produced. Very many cases are related of acute cynanche laryngea, successfully treated by the usual means of combating inflammation; but it may reasonably be doubted whether these measures would be efficacious in producing an absorption of serous fluid once effused, although they might arrest the progress of the disease, and thus prevent its being thrown out at all. Perhaps cases of sporadic disease which have commenced in the palate, the tonsils or the fauces, are those most likely to be benefited by such treatment; but it must be remarked, on the other hand, that in many of the cases which recovered without operation, it is distinctly stated that no trace of inflammation could be discovered on examining the throat." 99.

Mr. P. thinks it probable that, in the majority of these cases, the morbid action is seated in the subjacent cellular tissue, and then the effusion takes place with such rapidity that the disease is hardly formed until the mechanical obstruction is produced. As the means of reducing inflammation, after the effusion, will be of no use, our only hope must rest in procuring for the patient another channel than the larynx for the purpose of respiration. There are many reasons why bronchotomy should be promptly resorted to on such occasions. *First*, the hazard which is run by trying other means too long—*Secondly*, the operation allows the larynx to remain in a state of repose—. *Thirdly*, the danger of the wound is nothing, and temporary relief is certain, allowing more time for the surgeon to make an accurate examination of the case.

General blood-letting should be carried, in acute laryngitis, to the extent of producing syncope or relief of the symptoms, and then the system should be kept under the nauseating influence of tartar-emetic. But, if serous effusion has taken place in the submucous tissue, depletion will do no good, and may do harm, as the obstructed respiration, in itself, occasions a severe depression of the vital energy, through the medium of the brain. How are we to know whether the difficulty of breathing be occasioned by acute inflammation or effusion? If, after an impression has been made on the system by a full bleeding, we find no alleviation of the symptoms, it may afford presumptive evidence that the obstruction is mechanical, and prove an additional reason for resorting to the operation. If this be delayed beyond a certain period, and the following symptoms take place, bronchotomy can only afford some procrastination of the patient's fate, and a less painful death.

"It is very possible that a patient may perish during the first stage of this disease, being strangled by a spasmodic action of the muscles of the larynx, and thus die *constante mente integrisque sensibus:* indeed the unwillingness a patient feels to make an attempt to swallow, which always brings on a spasm, or sometimes even to speak, shows that he is sensible of the danger attendant on spasm, quite independent of the fearful distress it occasions. But it is not thus that the disease terminates in general, for after the difficulty of breathing has continued for some time, the patient's countenance becomes swollen and of a livid paleness,—the eyes are pearly white and suffused, as if an exhalation had taken place from the conjunctiva, and dried upon the membrane;—the lips are purple,—the disposition to slumber increases, whilst the extreme anxiety of the patient to maintain respiration, and his efforts to carry it on are increased. Every muscle that can be brought into action is forcibly employed, whilst the sound of the breathing is altered, and there is a kind of sob of distress accompanying each expiration. The sweat pours over the face and forehead, and perhaps from the entire body. Sometimes the patient becomes inconceivably restless: whilst again he may remain quiet, apparently under the influence of hopeless despondency. The pulse very generally increases in rapidity.

"After these symptoms have endured a few hours, the patient is observed to make less violent efforts to support respiration, and the intervals between each act are longer. The want of accordance between this function and circulation becomes more apparent; for the pulse is very small and encreased in frequency, and often immediately before death it is so rapid that its strokes cannot be counted. The breathing seems to be more a convulsive effort than a regular action, and is sometimes accompanied by stertor. The countenance becomes sunken, the eye loses its brilliancy still more, and the forehead is bedewed with a cold and clammy sweat. The patient becomes insensible, and death soon closes the scene." 105.

Two cases are related in illustration of this part of the subject, of which the following is the substance.

*Case* 5. A gentleman residing 16 miles from Dublin, was attacked with what he considered to be common sore throat. He had been affected in the evening with shivering, head-ache, pain in the throat, and some difficulty of deglutition. He passed an uneasy night, and awoke from a short sleep in a paroxysm of suffocation. By the time the family apothecary arrived, the symptoms had taken an alarming character. The patient was bled, and took purgatives. A large blister was applied to the throat; but no relief followed. Mr. P. happening to be in the neighbourhood, was called in, and found the patient with pallid swollen countenance—glassy and protruded eyes—loud, harsh, and stridulous breathing, with violent efforts to carry on this function—pulse rapid, but not full—senses perfect. He died in an hour afterwards, or 21 hours from the first approach of the disease.

On dissection, the lining membrane of the larynx appeared slightly inflamed, of a bright pink colour, but not thickened in structure. The epiglottis was unaffected. The submucous tissue was œdematous, so that the edges were approximated, and the passage nearly closed. The cavity of the larynx contained a good deal of frothy mucus, and its surface was smeared over with a yellowish glutinous substance, resembling honey. Permission was not granted to examine the lungs.

The following interesting case, by Dr. Bliss, published in the first volume of the Transactions of the Physico-Medical Society of New York, is one exactly in point, and we shall here quote it.

"William Potter, aged 28 years, a seaman, was admitted into the N. Y. Hospital on the 29th of December, 1814, with a fever of the remitting type. His skin was of a yellow colour, and bowels constipated. He had also slight catarrhal symptoms, with some soreness of the throat.

"He was convalescing from his fever, when, on the evening of the 13th of January, whilst the physician was visiting the ward, he complained of a sore throat, hoarseness, and cough. But these symptoms did not give rise to any particular alarm, on account of his previously having had an affection of the throat.

"The next morning, the house physician, Mr. Helme, being confined to his bed by illness, I was requested to see him.

"He complained of extreme difficulty of breathing, and had the peculiar wheezing and cough of croup. His voice was remarkably shrill, and it was with great difficulty he could articulate. He referred to the larynx, as the seat of his distress. The fauces were slightly inflamed; but deglutition was performed with very little difficulty. The pulse was frequent and full, but not corded. The skin hot, and the tongue covered with a white fur. The face rather flushed.

"His symptoms appearing so exceedingly urgent, the jugular vein was opened, and about twenty ounces of blood drawn. An emetic was given, with a view of vomiting him; and although the patient took to the amount of three drachms of ipecacuana, and twenty grains of emetic tartar, in divided doses; and notwithstanding mechanical irritation of the fauces was repeatedly employed, very little vomiting was excited. Little more was ejected, than the drinks which he had taken, to quicken the operation of the emetic medicine. The operation of vomiting, however, had the effect of producing a moist state of the skin, and of rendering respiration rather less difficult.

"After this, a piece of flannel, dipped in hot aqua ammoniæ, was applied to the anterior part of the neck, which soon caused vesication of the skin. The patient was also directed to inhale the vapour of hot vinegar and water.

"About 11 o'clock, the emetic medicine produced several very free evacuations from the bowels; the pulse, at this time, was very much reduced. The difficulty of breathing however, was not in the slightest degree diminished, but exceedingly distressing to witness. At this time a large epispastic was applied to the upper part of the chest.

"Between 12 and 1 o'clock, I was again summoned to the ward, and found the patient apparently in the agonies of death. The respiration horridly difficult; the countenance livid and shrunk; and the eyes as it were, starting from their sockets. This paroxysm increased, till the patient was seized with convulsions. The pulse ceased to beat, and respiration was momentarily suspended. From this state he recovered in a slight degree. At this time, the operation of tracheotomy was proposed; and the assent of Dr. Hamersley, the attending physician, having been obtained, I immediately performed it: being assisted by Mr. Inderwick, of the United States' navy.

"During the operation, the patient had another violent paroxysm of difficult respiration; and, to the by-standers, appeared to be dying. A gum elastic tube was introduced into the opening, and after the blood, which had escaped from the wound, had been thrown from the trachea, the patient breathed with tolerable ease.

"It became necessary several times in the course of the afternoon, to remove the tube, and introduce a fresh one; on account of its becoming obstructed by the mucus excreted from the trachea.

"A decoction of seneka was now directed for the patient: but every attempt at swallowing was followed by a fit of coughing, and his drinks appeared to find their way into the trachea. On examination of the throat, it appeared that the epiglottis was thrown back towards the apex of the tongue, so that the larynx was not perfectly closed in the act of deglutition. The patient at times breathed with considerable ease, through the afternoon and evening; at other times, from the mucus becoming accumulated, the respiration was rendered more difficult, till relieved by a fit of coughing. In the forepart of the evening, a vapour bath was employed for his relief. But although the operation had evidently prolonged his existence, yet it appeared the disease was not subdued, and we were doomed to witness the inefficacy of our remedies. The patient expired about fifteen minutes after 10 o'clock in the evening.

"On dissection, it was observed, that beneath the muscles covering the larynx, there was a slight effusion of coagulable lymph. The upper part of the pharynx and the fauces exhibited traces of inflammation. The epiglottis gave evidence of having been highly inflamed. Beneath the membrane covering this part, on either side, there was a considerable effusion of serum, in distinct cavities or sacs.

"This effusion was so considerable, as to revert the epiglottis, and prevent its closing the glottis. This, as well as other parts of the larynx, was covered, on its internal surface, by a number of dark-coloured spots, which gave it a mottled appearance.

"The whole of the larynx and trachea showed a high degree of inflammation, on their internal surfaces. The mucous membrane, lining the larynx, was remarkably thickened, and beneath it there was a very considerable serous effusion. So considerable was this thickened state of the membrane and the effusion beneath, that the rima glottidis was almost completely closed.

"This circumstance, probably, gave rise to the symptoms of suffocation, which occurred previous to the operation having been performed. Besides these appearances, there was a considerable quantity of tenacious mucus adhering to the inner surface of the wind-pipe throughout its course." 124.

The second case was not seen during life. The patient, a lad of 19 or 20, had been ill only one day, and was supposed to have died of poison. But, on examination, the rima glottidis was found completely closed by œdema of the sub-mucous tissue, the result of acute inflammation, for the epiglottis was scarlet, and swollen to twice its natural size.

The remaining half of the volume is occupied with chronic diseases of the larynx, including laryngeal phthisis—foreign bodies introduced into the air-passage—wounds of the larynx and trachea—and observations on the operation of bronchotomy itself. These will form the subject of a separate article for our next number, in which we shall introduce some additional information on these important topics. In the mean time, we have exhibited sufficient specimens of the work to enable the public to judge of its nature and merits. To the junior practitioner, the work certainly affords a very useful manual on the class of diseases investigated—and to practitioners in general it may be no unimportant object to have a distinct work on diseases, accidents, and operations, in such parts as the larynx and trachea.

